# Serum levels of osteoprotegerin and receptor activator of nuclear factor -κB ligand in children with early juvenile idiopathic arthritis: a 2-year prospective controlled study

**DOI:** 10.1186/1546-0096-8-30

**Published:** 2010-12-06

**Authors:** Gunhild Lien, Thor Ueland, Kristin Godang, Anne M Selvaag, Øystein T Førre, Berit Flatø

**Affiliations:** 1 Department of Rheumatology, Oslo University Hospital, Rikshospitalet, Norway; 2 Department of Rheumatology, Diakonhjemmet Hospital, Oslo, Norway; 3 Section of Endocrinology, Medical Department, Oslo University Hospital, Rikshospitalet, Norway; 4 Research Institute for Internal Medicine, Oslo University Hospital, Rikshospitalet, Norway

## Abstract

**Background:**

The clinical relevance of observations of serum levels of osteoprotegerin (OPG) and receptor activator of nuclear factor -κB ligand (RANKL) in juvenile idiopathic arthritis (JIA) is not clear. To elucidate the potential role of OPG and RANKL in JIA we determined serum levels of OPG and RANKL in patients with early JIA compared to healthy children, and prospectively explored changes in relation to radiographic score, bone and lean mass, severity of the disease, and treatment.

**Methods:**

Ninety children with early oligoarticular or polyarticular JIA (ages 6-18 years; mean disease duration 19.4 months) and 90 healthy children individually matched for age, sex, race, and county of residence, were examined at baseline and 2-year follow-up. OPG and RANKL were quantified by enzyme-immunoassay. Data were analyzed with the use of t-tests, ANOVA, and multiple regression analyses.

**Results:**

Serum OPG was significantly lower in patients than controls at baseline, and there was a trend towards higher RANKL and a lower OPG/RANKL ratio. Patients with polyarthritis had significantly higher increments in RANKL from baseline to follow-up, compared to patients with oligoarthritis. RANKL was a significant negative predictor for increments in total body lean mass. Patients who were receiving corticosteroids (CS) or disease-modifying antirheumatic drugs (DMARDs) at follow-up had higher OPG/RANKL ratio compared with patients who did not receive this medication.

**Conclusions:**

The data supports that levels of OPG are lower in patients with JIA compared to healthy children, and higher levels of RANKL is associated with more serious disease. RANKL was a significant negative predictor of lean mass in patients with JIA. The OPG/RANKL ratio was higher in patients on DMARDs or CS treatment.

## Background

Children with juvenile idiopathic arthritis (JIA) are at risk of bone destructions and reduced bone mass. The pathogenesis for the bone loss is complex and is influenced by inflammation, physical inactivity, nutrition and medication. The immune and skeletal systems share a number of regulatory molecules, and there is accumulating evidence indicating interactions between the two systems [1].

Bone remodelling is a lifelong continuous process conducted by osteoblasts, synthesizing bone matrix and its resorption by osteoclasts. Important regulators of osteoclast recruitment and function are the three key molecules Osteoprotegerin (OPG), Receptor Activator of Nuclear factor -κB (RANK) and its ligand (RANKL). RANKL stimulates osteoclast production and survival via the membrane -bound receptor RANK, [2-5] while OPG inhibits osteoclast differentiation and activation due to its function as a non-signalling decoy receptor for RANKL [6]. The physiological balance between RANKL and OPG is regulated by various calcitropic cytokines and hormones and alterations in their ratio are critical in the pathogenesis of bone diseases [7]. Osteoblasts and T cells are important producer cells of RANKL. An inflammatory environment with T-cell activation may tilt the balance between OPG and RANKL and increase osteoclast activation and bone resorption.

In patients with early rheumatoid arthritis (RA), baseline serum OPG/RANKL ratio and inflammation have independently predicted radiographic progression of joint damage [8]. RANKL-expressing cells and RANK-expressing osteoclast precursor cells, and more limited OPG, have been demonstrated at sites of subchondral bone erosions [9]. In children with JIA, over expression of RANKL has been detected in synovial fluid mononuclear cells from joints [10]. Higher serum RANKL and lower serum OPG/RANKL ratio has been found in two studies of patients with JIA compared to controls [11,12], while higher serum levels of OPG [13,14] or an increased OPG/RANKL ratio [14] has been shown in other studies. Publications concerning this topic in patients with JIA are still few, the studies cross-sectional, and the clinical relevance of the observations is not clear.

To further elucidate the potential role of OPG and RANKL in JIA we prospectively explored serum levels of OPG and RANKL in an observational cohort study of children with early disease, compared to individually matched children, and in relation to radiographic score, bone and lean mass, disease activity, and medication.

## Methods

### Study participants

All Caucasian children with JIA between the ages of 6 and 18 years who were attending the Department of Rheumatology, Oslo University Hospital, Rikshospitalet, for the first time from May 1995 to February 1999 were invited to participate in a two-year prospective study of bone mass and bone turnover. Of 127 eligible patients, 108 (85%), living in 16 different counties (latitudes from 58°N to 68°N) were included. Each patient was individually matched to a healthy child with the same sex, age, race, and county of residence who was randomly selected from the National Population Register. The characteristics of the total patient group, including nutritional status, level of physical activity, markers of bone formation and bone resorption, and bone mass, have previously been described [15]. In the present study we included only patients who met the JIA criteria [16] for oligoarthritis (n = 59) or polyarthritis (n = 31) and their controls (n = 90). Patients' mean disease duration was 19.4 months (SD 12.3). The participants were examined at baseline and at follow-up, a mean of 24.2 months (SD 1.4) later. The Regional Ethics Committee for Medical Research approved the study. Written informed consent was obtained from the parents and from children older than 16 years.

### Clinical examination

Clinical information was obtained by interviews, physical examination and questionnaires [15,17-22].

### Radiographic examination

Radiographs of the non-dominant hand and wrist were taken for assessment of skeletal maturity of all study subjects at baseline and follow-up [23]. Radiographs of the patients' knees and ankles were obtained at the time of admission to hospital; other joints when clinically indicated, and were scored by one of 2 trained radiologists (KD and VJ) according to a radiographic classification system for juvenile rheumatoid arthritis: grade 0 (normal joints), grade 1 (juxtaarticular osteoporosis and/or periarticular soft tissue swelling), grade 2 (growth abnormality, bony erosion not present), grade 3 (growth abnormality and marginal bony erosions), grade 4 (deformation and severe erosions), and grade 5 (gross destruction and deformity) [24,25]. Radiographic progression was defined as an increase in the radiographic grade during the study. An increase from grade 0 to 1 was not considered to be radiographic progression.

### Laboratory measures

Venous blood samples were obtained before noon at baseline and follow-up. Bone markers were measured in the second void urine sample of the morning. For analysis of OPG, RANKL and C-reactive protein (CRP) serum were stored at -70°C until analyzed. OPG and CRP (detection limit 0.16 mg/L) was quantified by enzyme immunoassays (EIA) using commercially available matched antibodies from R&D systems (Minneapolis, Minnesota) [26] and Dako cytomation (Glostrup, Denmark), [27] respectively. RANKL was quantified by EIA (Bender MedSystems, Vienna, Austria). Bone formation was assessed by serum levels of bone-specific alkaline phosphatase and osteocalcin, bone resorption by serum levels of C-telopeptide of type 1 collagen and urinary concentration of deoxypyridinoline, [28-30] and vitamin D stores by serum concentration of 25-hydroxyvitamin D and the active hormonal metabolite by serum 1,25-dihydroxyvitamin D3, all samples analyzed immediately by routine laboratory methods.

### Bone mass measurements

Measurements of the total body, distal radius, femoral neck and L2-L4 of the lumbar spine were evaluated with the same dual x-ray absorptiometry (DXA) equipment (Lunar Expert-XL; GE Lunar, Madison, WI). All analyses were performed by one investigator using Expert-XL software version 1.72 and 1.91 and were read by one investigator (GL). The bone mineral content (BMC) was calculated from all the regions. Fat and lean composition of soft tissue was calculated from the total body scan. The long-term precision of the scanner was tested daily with minimal drift in the measurements and a coefficient of variation (CV) of 0.5%. The in vivo precision of the operator technique (patients and healthy subjects) was 1.6% for lumbar spine and 2.0% for femoral neck [31]. The total body BMC findings were calculated as Z-scores in terms of the number of standard deviations (SD) above or below the age-specific mean for healthy individuals (Z-score = [subject's measurement - mean measurement of the reference population]/SD of the reference population). By definition, 16% of healthy children will have a Z-score less than -1.0 SD, and 2.3% will have a Z-score less than -2.0, but no evidence-based guidelines exist to define osteopenia and osteoporosis in children [32]. We defined low BMC as Z-score between -1 SD and -2 SD and very low BMC as a Z-score > 2 SD below the mean.

### Statistical analysis

Differences between patients and matched controls for clinical, radiographic, bone mass and laboratory measures were tested by paired samples t-test for continuous variables and McNemar's test for categorical variables. Within the patient cohort, differences were tested by the independent samples t-test or by one-way analysis of variance using the Bonferroni correction for multiple comparisons for continuous variables, and the chi-square test for categorical variables.

Multiple regression analyses were performed to identify predictors of the 2-year changes in body lean mass, BMC and numbers of active or mobility restricted joints. Explanatory variables were included in the model if the p value was less than 0.2 in unadjusted linear regression analyses or if a variable was known to be associated with the outcome variable [33]. Highly intercorrelated independent variables (r > 0.7) in a multiple model were avoided. To reduce the possibility of body size-related artefacts in the analyses of bone mass, bone area, weight, and height were included in the multiple regression models for BMC [34]. Forward stepwise regression methods were used.

For all analyses, p values less than or equal to 0.05 (2-tailed tests) were considered significant. The statistical analysis was performed using SPSS software version 14.0 (SPSS, Chicago, IL).

## Results

### Demographic and clinical data

A total of 90 JIA patients and 90 controls were included. Clinical features at baseline and 2-year follow-up are shown in table 1.

**Table 1:** Characteristics of patients with JIA and healthy children at baseline and 2-year follow-up*

	Baseline		2 year follow-up	
	
	JIA patients(n = 90)	Controls(n = 90)	JIA patients(n = 90)	Controls(n = 90)
Characteristics				
Female, n (%)	56 (62%)	56 (62%)	56 (62%)	56 (62%)
Age, mean (SD) years	10.1 (3.2)	10.1 (3.2)	12.1 (3.2)	12.2 (3.2)
Disease-onset type, n (%)				
Oligoarthritis, persistent	48 (53%)	-	48 (53%)	-
Oligoarthritis, extended	11 (12%)	-	11 (12%)	-
Polyarthritis, RF negative	28 (31%)	-	28 (31%)	-
Polyarthritis, RF positive	3 (3%)	-	3 (3%)	-
Disease duration, mean (SD) months	19.4 (12.3)	-	44.3 (14.2)	-
No. of joints with active disease, mean (SD) †	2.2 (3.5)	-	1.3 (4.2)	-
No. of joints with restricted mobility, mean (SD) †	1.7 (2.6)	-	1.2 (2.2)	-
Radiographic score, mean (SD) ‡	1.7 (0.6)	-	1.8 (0.6)	-
Radiographic erosions, n (%) §	3 (3%)	-	5 (6%)	-
Radiographic progression, n (%)¶	-	-	9 (10%)	-
Total body BMC Z-score < -1, n (%)	10 (11%)	9 (10%)	20 (22%)	12 (13%)
Disease-modifying antirheumatic drugs, current use, n (%)	48 (53%)	-	37 (41%) #	-
Non-steroidal anti-inflammatory drugs, current use, n (%)	68 (76%)		43 (48%)	
Oral corticosteroids **				
Current use, n (%)	17 (19%)	-	8 (9%)	-
Dosage, mean (SD) mg/day	11.2 (7.1)	-	6.7 (5.9)	-
Ever used, n (%)	27 (30%)	-	31 (34%)	-
Cumulative dose, mean (SD) mg	1234 (1328)	-	1919 (2062)	-
Cumulative dose, mean (SD) mg/kg of body weight	38 (42)	-	52 (65)	-

* Because of rounding, not all percentages total 100. JIA = juvenile idiopathic arthritis; RF = rheumatoid factor; BMC = bone mineral content.

† Sixty-nine joints were evaluated.

‡ Highest registered score from a range of 0 = best to 5 = worst.

§ Erosions = radiographic grade 3 or higher.

¶ Increase in radiographic grade from baseline to follow-up.

# Methotrexate (n = 24), hydroxychloroqine (n = 8), methotrexate plus hydroxychloroqine (n = 3), methotrexate plus sulfasalazine (n = 1) and methotrexate plus etanercept (n = 1).

** Prednisolone. None of the patients had taken methylprednisolone.

### Serum levels in JIA patients and controls

**Figure 1:**
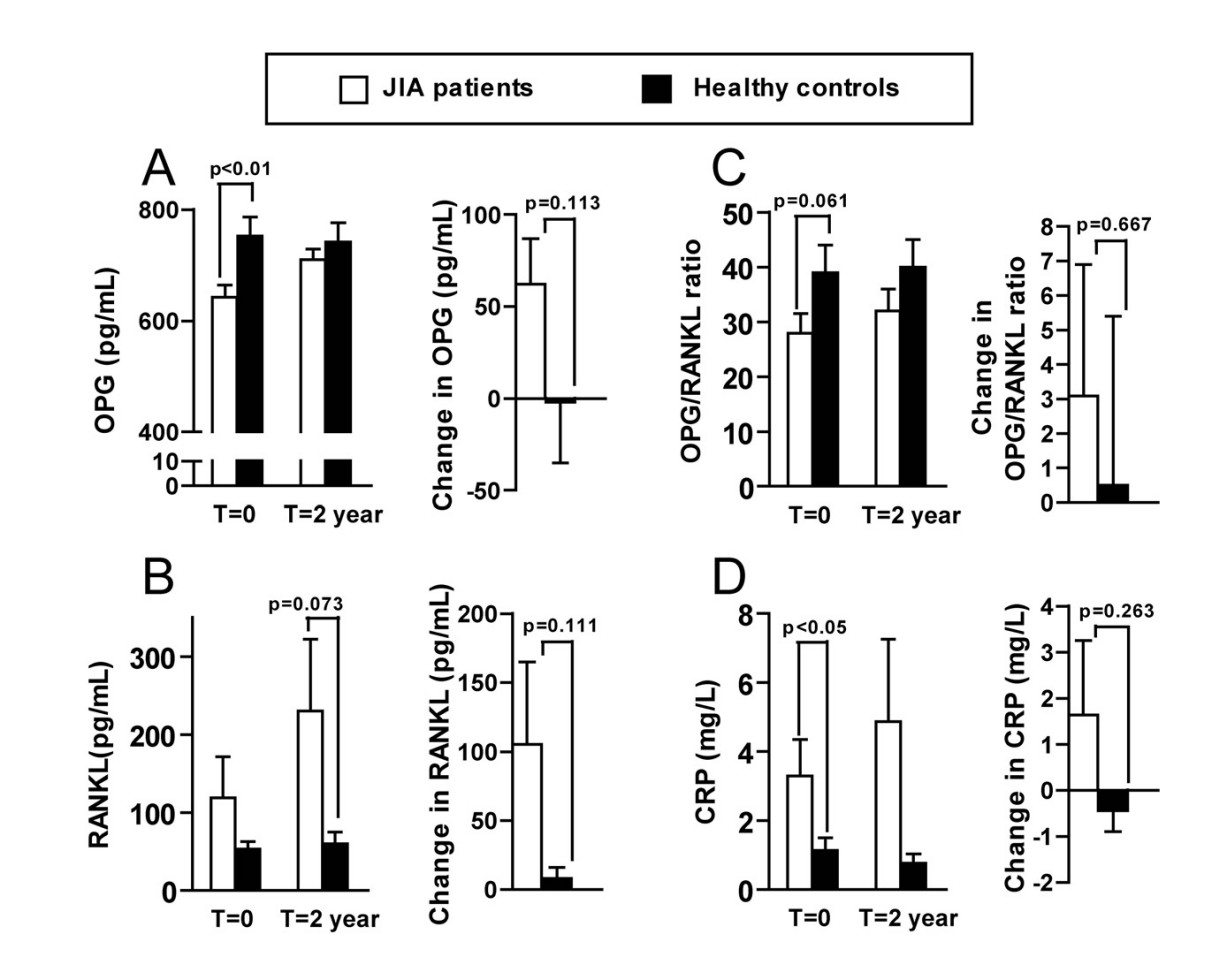
**Serum levels in patients with JIA and controls at baseline and 2-year follow-up**. A. OPG. B. RANKL. C. OPG/RANKL ratio. D. CRP. p Values by paired samples t-test for patients vs controls. Values are the mean. Error bars represent standard error of the mean.

Figure 1 shows the serum levels and changes from baseline to 2-year follow-up in patients and controls. The OPG level was significantly lower in the patients than in the controls at baseline (p = 0.003). The levels of RANKL were higher in the patients at follow-up (p = 0.073), but the findings did not reach statistical significance. The OPG/RANKL ratio tended to be lower in the patients than controls at baseline and follow-up (p = 0.061 and p = 0.200). The level of CRP was significantly higher in the patients at baseline (p = 0.036). There were no significant differences in serum levels of OPG, RANKL, OPG/RANKL ratio or CRP between males and females (data not shown).

### Changes in serum levels, radiographic score and bone mass

**Figure 2:**
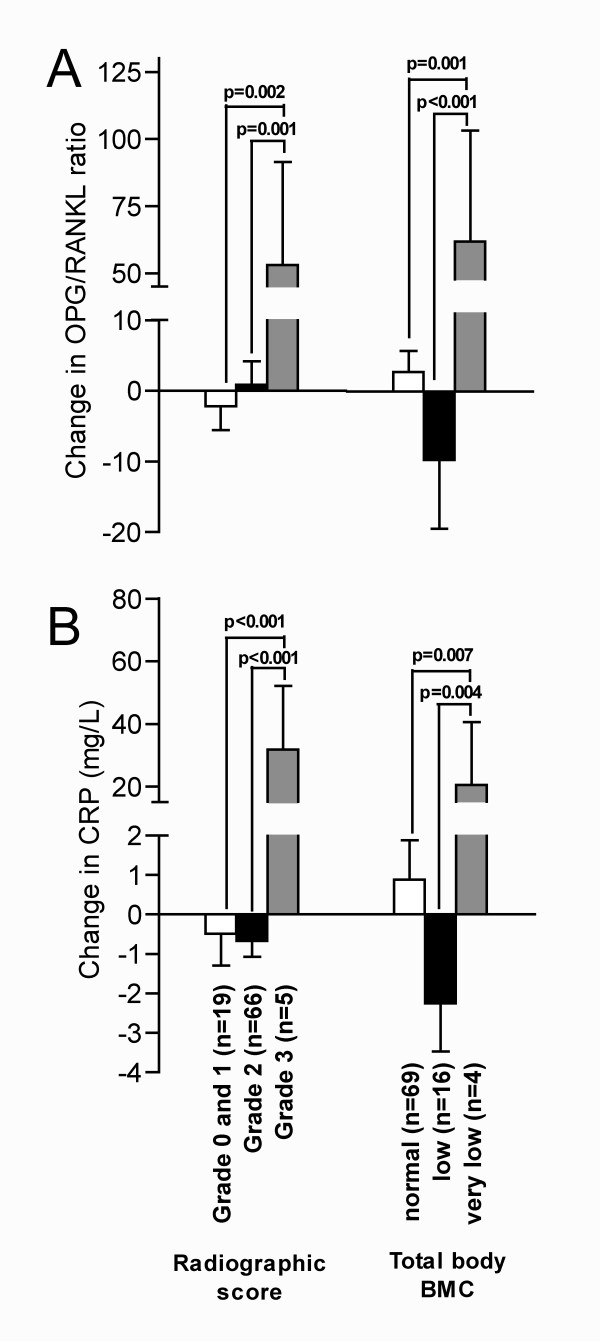
**Changes in serum levels, radiographic score and total body BMC at 2-year follow-up in patients with JIA**. Changes in serum levels from baseline to follow-up: A. OPG/RANKL ratio. B. CRP. The legends for radiographic score and total body BMC is valid for panel A and B. Radiographic score grade 0 = normal joints; grade 1 = swelling/osteopoprosis; grade 2 = abnormal growth; grade 3 = abnormal growth and erosions; BMC Z-score > -1 SD defined as normal; BMC Z-score between -1 SD and -2 SD defined as low; BMC Z-score < -2 SD defined as very low. p Values by one-way analysis of variance, using the Bonferroni correction for multiple comparisons within the groups. Values are the mean. Error bars represent standard error of the mean.

Changes in the OPG/RANKL ratio and serum levels of CRP from baseline to 2-year follow-up, in relation to different radiographic scores and total body BMC Z-scores, are shown in Figure 2. No patient had higher radiographic score than grade 3 with growth abnormality and bony erosions. The increments from baseline to follow-up of the OPG/RANKL ratio and CRP were significantly higher in patients with very low BMC and patients with erosions, compared to patients with less serious findings (p values ranged from <0.001 to 0.007). A greater increase in OPG/RANKL ratio was also seen in patients with radiographic progression compared to the patients without radiographic progression (difference of increase 33.7, 95%CI = 10.7, 56.7, p = 0.004).

### Changes in serum levels and disease-onset type

**Figure 3:**
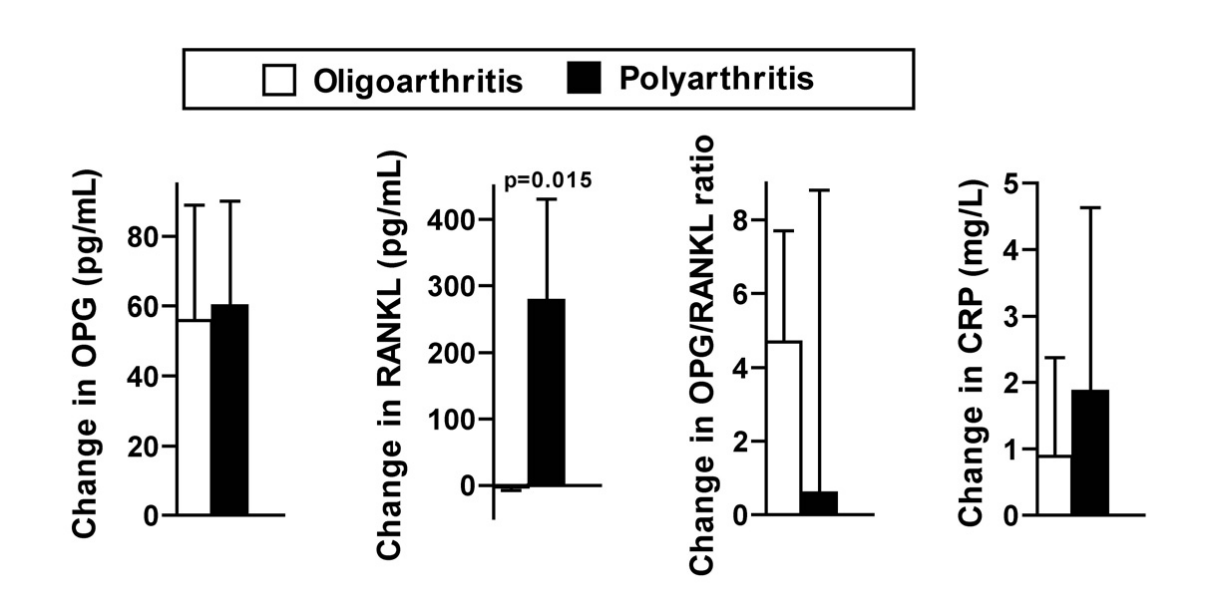
**Changes in serum levels in patients with oligoarthritis (OA) and polyartrhritis (PA)**. Changes from baseline to 2-year follow-up: OPG, RANKL, OPG/RANKL ratio and CRP. p Values by independent samples t test for patients with OA vs PA. Values are the mean. Error bars represent standard error of the mean.

The levels of RANKL increased significantly more from baseline to follow-up in the polyarthritis-group than in the oligoarthritis-group (p = 0.015), while the changes in OPG, OPG/RANKL ratio and CRP were not significantly different (Figure 3).

### Serum levels, antirheumatic and corticosteroid treatment

**Figure 4:**
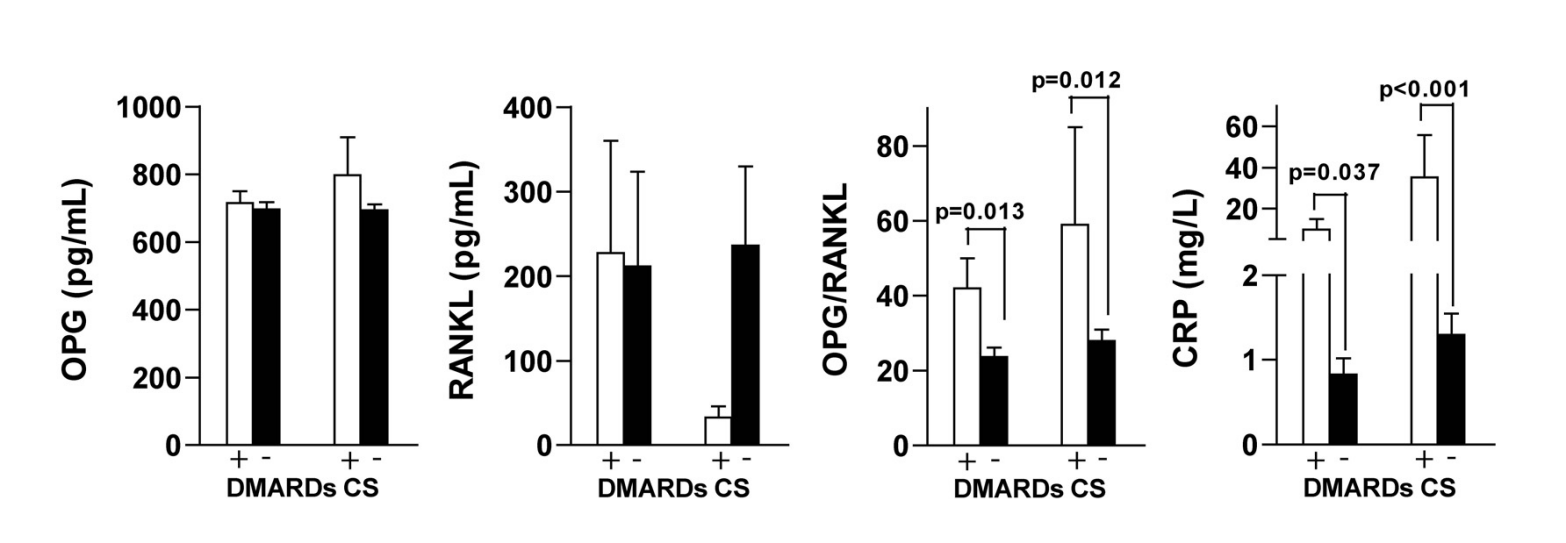
**Serum levels and DMARDs and CS at 2-year follow-up in patients with JIA**. Serum levels of OPG, RANKL, OPG/RANKL ratio and CRP. p Values by independent samples t test for current treatment with DMARD or CS vs not. Values are the mean. Error bars represent standard error of the mean.

Figure 4 shows serum OPG, RANKL, OPG/RANKL ratio and CRP at 2-year follow-up in relation to current DMARD and corticosteroid treatment. The levels of the OPG/RANKL ratio and CRP was significantly higher in the patients who were currently treated with DMARDs compared to the patients who were not (p = 0.013 and p = 0.037). Analyses comparing DMARD subgroups did not show significant differences. The serum OPG/RANKL ratio and CRP were significantly higher as well in patients who were current users of corticosteroids compared to those who were not (p = 0.012 and p < 0.001).

### Predective value of OPG, RANKL, OPG/RANKL ratio and changes in lean mass, bone and affected joints

Laboratory measurements, patient and disease characteristics, physical activity and medication assessed at baseline were explored as independent predictors of the 2-year changes in lean mass, BMC and no. of active or mobility restricted joints by unadjusted linear regression analysis. Predictors chosen for the multiple regression model of the change in total body lean mass were baseline weight, height, bone age, weight-bearing physical activity, serum ionized calcium, 25-hydroxyvitamin D, parathyroid hormone, bone-specific alkaline phosphatase, C-telopeptide type 1, urinary deoxypyridinoline, baseline OPG, RANKL and CRP. In the final regression model baseline RANKL was a significant independent negative predictor of increased lean mass at follow-up (regression coefficient -1.5, 95% CI = -2.88,-0.12, p = 0.034) together with the independent positive predictors weight-bearing physical activity (regression coefficient 216, 95% CI = 51, 381, p = 0.011), bone-specific alkaline phosphatase (regression coefficient 31.7, 95% CI = 10.7, 52.6, p = 0.004) and 25-hydroxyvitamin D (regression coefficient 38.9, 95% CI = 0.48, 77.3, p = 0.047). Serum levels of OPG, RANKL or the OPG/RANKL ratio were not identified as independent predictors of the changes in BMC of total body, distal radius, femur neck or lumbar spine, or the changes of no. of joints with active disease or restricted mobility (data not shown).

## Discussion

In the present prospective, observational cohort study of 90 JIA patients, early in the disease course, the level of OPG was significant lower than in 90 matched healthy children. Furthermore, during 2-years follow-up, RANKL increased more in patients with polyarthritis than in patients with oligoarthritis and RANKL was a significant negative predictor of lean mass. The OPG/RANKL ratio at follow-up was higher in patients on DMARD or CS treatment. To our knowledge, this is the first prospective controlled study of OPG and RANKL in children with JIA.

The findings of lower serum levels of OPG in our JIA patients with early disease are consistent with findings in children with untreated juvenile dermatomyositis [35], and so are the trends towards higher levels of RANKL and a lower OPG/RANKL ratio. Other studies exploring serum OPG and RANKL in JIA, differs from ours, but consist of patients with longer disease duration [11,12,14], more severe disease [12,14] or different subgroups [11], complicating comparisons. A recent study of polyarticular JIA patients showed higher serum levels of RANKL, a lower OPG/RANKL ratio, and comparable OPG levels compared to controls [12]. Another recent study, with a mixture of children and adults, found similar results in oligoarticular and polyarticular subtypes of JIA compared to controls [11]. A previous study has shown higher serum OPG levels and a higher OPG/RANKL ratio in oligoarticular and polyarticular JIA patients compared to controls, in contrast to the other studies [14]. These differences may have several explanations. As mentioned, the composition, age range and disease severity is not necessarily comparable between the studies. Importantly, we measured free RANKL and immunoassays measuring both OPG bound and free RANKL may give different results. An increase in circulating OPG has often been viewed as a compensatory response [14]. However, members of the TNF ligand superfamily often circulate at low levels with a short half time. Thus, as seen for other soluble TNF receptors [36], OPG may represent a reliable marker of the overall activity of the OPG/RANK/RANKL axis as well as a stable marker of inflammation. The correlation between CRP and the OPG/RANKL ratio in our JIA cohort supports this.

We found that baseline RANKL was a significant and independent negative predictor and weight-bearing physical activity a positive predictor of the gain in total body lean mass in the JIA patients. There are few data elucidating the impact of RANKL on the variation in lean mass in children but our results are most likely related to the chronic inflammation. In adult RA patients cachexia with muscle wasting and fat gain is common, and the mechanisms probably include cytokine-driven hypermetabolism during active disease [37,38].

The molecular pathways and specific effects of conventional DMARDs on bone and cartilage are not clearly defined [39]. However, DMARDs may have an effect on the osteoclast formation. In a study of cultures of fibroblast-like synoviocytes from patients with RA, the DMARDs methotrexate and sulfasalazine, have been shown to decrease the ratio of RANKL/OPG in a dose-dependent manner [40]. Another study of synovial tissue from patients with RA, treated with DMARDs, has shown increased OPG expression and decreased RANKL expression [41]. Our results with an increased OPG/RANKL ratio in DMARDs treated and corticosteroid treated patients are in accordance with these findings. Although corticosteroids are generally not considered conventional DMARDs there is evidence that corticosteroids have structure-sparing effects and can reduce the rate of erosion progression in RA [39,42].

There have been limited knowledge of the disease course during the first years of JIA and this study was part of a larger prospective comprehensive study [43]. Our patients were comparable to JIA patients in epidemiologic studies [44]. They were explored early in the disease course, the mean disease activity was low, the numbers of patients with radiographic erosions (6%) were low, and the numbers of patients with very low bone mass (4%) were low. The overall low disease activity in our patients is however a limitation for the interpretation of the results and the present study seems somewhat underpowered in places with strong trends but statistical significance not being attained. If we had chosen a patient cohort with higher disease activity and longer disease duration, the numbers of patients with joint erosions and very low bone mass would more likely have been higher. In addition, if more sensitive imaging methods as ultrasonography and magnetic resonance imaging (MRI), had been available as supplements to conventional radiographs, we might have detected a higher number of patients with structural damage [45,46].

## Conclusions

In summary, the JIA patients with oligo- or polyarthritis had significant lower levels of OPG early in the disease course compared to controls. The patients tended to have higher levels of RANKL and a lower OPG/RANKL ratio consistent with earlier findings. Baseline RANKL was a significant negative predictor of total body lean mass. To better understand bone loss and the clinical significance of the balance between OPG and RANKL in children with JIA more prospective data are warranted. Inclusion of children early in the disease course and before treatment with oral corticosteroids, DMARDs or biologic therapy will add information to our knowledge.

## Abbreviations

BMC: Bone mineral content; CS: Corticosteroids; CRP: C-reactive protein; DMARDs: disease-modifying antirheumatic drugs; DXA: dual x-ray absorptiometry; JIA: juvenile idiopathic arthritis; OPG: Osteoprotegerin; RANKL: receptor activator of nuclear factor -κB ligand; TNF: tumor necrosis factor

## Competing interests

The authors declare that they have no competing interests.

## Authors' contributions

GL contributed to conception and design, acquisition, analysis and interpretation of data, drafting of manuscript and final approval of manuscript. TU contributed to conception and design, acquisition, analysis and interpretation of data, revision of manuscript and final approval of manuscript. KG, AMS and BF contributed to conception and design, acquisition and analysis of data, revision of manuscript and final approval of manuscript. OF contributed to conception and design, revision of manuscript and final approval of manuscript.
